# M-charts as a tool for quantifying metamorphopsia in age-related macular degeneration treated with the bevacizumab injections

**DOI:** 10.1186/1471-2415-13-13

**Published:** 2013-04-15

**Authors:** Katarzyna Nowomiejska, Agnieszka Oleszczuk, Agnieszka Brzozowska, Andrzej Grzybowski, Katarzyna Ksiazek, Ryszard Maciejewski, Piotr Ksiazek, Anselm Juenemann, Robert Rejdak

**Affiliations:** 1 Department of General Ophthalmology, Medical University, Lublin, Poland; 2 Department of Mathematics and Medical Biostatistics, Medical University, Lublin, Poland; 3 Department of Ophthalmology, Poznań City Hospital, Poznan, Poland; 4 Medical Faculty, University of Warmia and Mazury, Olsztyn, Poland; 5 Human Anatomy Department, Medical University, Lublin, Poland; 6 Department of Public Health, Medical University, Lublin, Poland; 7 Department of Ophthalmology, University of Erlangen-Nürnberg, Erlangen, Germany; 8 Centre of Ophthalmology, Institute for Ophthalmic Research, University of Tuebingen, Tuebingen, Germany; 9 Department of Experimental Pharmacology, Medical Research Centre, Polish Academy of Sciences, Warsaw, Poland

**Keywords:** Age-related macular degeneration, M-charts, Amsler grid, Metamorphopsia, Intravitreal injections

## Abstract

**Background:**

This article is aimed to assess quantitatively metamorphopsia using M-charts in patients suffering from wet age-related macular degeneration (AMD) treated with the intravitreal bevacizumab injections and to compare the results with traditional Amsler grid and ocular coherence tomography (OCT).

**Methods:**

Thirty-six patients diagnosed with wet AMD were examined one day before and one month after the intraocular injection of bevacizumab. Horizontal and vertical metamorphopsia scores using M-charts, distance visual acuity, Amsler test and OCT were performed at each visit. Additionally, 23 healthy subjects were examined as a control group.

**Results:**

The rate of metamorphopsia detection was 89% with M-charts and 69% with Amsler test. The horizontal metamorphopsia score improved in 22 patients, the vertical metamorphopsia score improved in 16 patients, the Amsler grid results improved in 6 patients, visual acuity improved in 17 patients. There was no correlation between the degree of metamorphopsia and the visual acuity or the central retinal thickness (CRT). The specificity of both the M-charts and Amsler grid was 100%.

**Conclusions:**

The rate of metamorphopsia detection in wet AMD patients was better with M-charts than with Amsler grid. M-charts may be used in the assessment of efficacy of treatment with intravitreal bevacizumab injections as another outcome measure, moreover they can be used even at home for the self-assessment. M-charts provide additional information concerning the visual function, independent of the visual acuity, CRT and morphological changes in OCT.

## Background

Metamorphopsia is a hallmark sign in patients with macular diseases and can be defined as a deformation of seen straight lines due to the displacement of photoreceptors. Age-related macular degeneration (AMD) is the most common macular disease affecting millions of aged people in the developed countries [1]. The most effective method of treating wet AMD is currently the anti-vascular endothelial growth factor (VEGF) – bevacizumab or ranibizumab [1]. With introduction of new treatment modalities preserving macular function, non-invasive and quick assessment of efficacy of the treatment is crucial for diagnostics of AMD. Fluorescein angiography may be associated with serious complications [2], hence it has been replaced in clinical practice by Optical Coherence Tomography (OCT). OCT is proving to be an accurate and reproducible tool for qualitative and quantitative assessment of the macular structure [3]. For assessment of the visual function, visual acuity and Amsler grid have been the gold standard.

The Amsler grid, consisting of evenly spaced horizontal and vertical lines, has been widely used since 1947 to test the metamorpho psia [4]. The Amsler grid is very cheap, straightforward and easily understood by the patient. However, it also produces high false negative rate [5]. Moreover, this test does not allow for the quantification of the severity of metamorphopsia; thus, it is difficult to monitor the visual function over time and to evaluate the effectiveness of treatment with anti-VEGF agents.

The M-chart (Inami Co., Tokyo, Japan) is a diagnostic tool developed by Matsumoto [6] to quantify the degree of metamorphopsia in patients with macular diseases. The usefulness of M-charts has been already shown in patients with epiretinal membranes [7,8], macular holes [9] and branch retinal vein occlusion [10].

The aim of this study was to evaluate metamorphopsia quantitatively, using the M-charts in patients suffering from wet AMD before and after bevacizumab injection and to compare the results with traditional Amsler grid and OCT results.

## Methods

Thirty-six patients (12 men, 24 women) were selected prospectively from the retinal out-patient clinic of the Department of General Ophthalmology in Lublin, Poland. The study was approved by the independent ethics committee and performed in accordance with the Declaration of Helsinki. Written informed consent was taken from each participant after the explanation of the nature of the study had been provided. Patients with wet AMD, diagnosed by means of OCT and with the best corrected visual acuity, better than or equal to 0.1, were included in the study. The exclusion criteria were diseases affecting the retina, other than AMD, such as glaucoma, amblyopia, strabismus, prior intraocular surgery, refracting error greater than ±5D.

The baseline examination and the follow-up examination performed one month later included a complete ophthalmological examination (slit lamp examination, applanation tonometry, dilated funduscopy) and OCT (Cirrus, Zeiss). Visual function testing included: best corrected distance visual acuity test using Snellen charts and the standard Amsler chart (black grid on a white background) with near correction and undilated pupils. The M-charts were used to assess both the horizontal and vertical metamorphopsia scores. The M-charts consist of 19 dotted (dot size is 0.1°) lines with dot intervals ranging from 0.2° to 2.0° of visual angle. In the centre of each line, there is a fixation point of 0.3°. The examination is performed at the distance of 30 cm with appropriate near correction. Dotted lines with interval changes from fine to coarse are printed on the following paper pages and are shown to the patients one after another.

**Figure 1:**
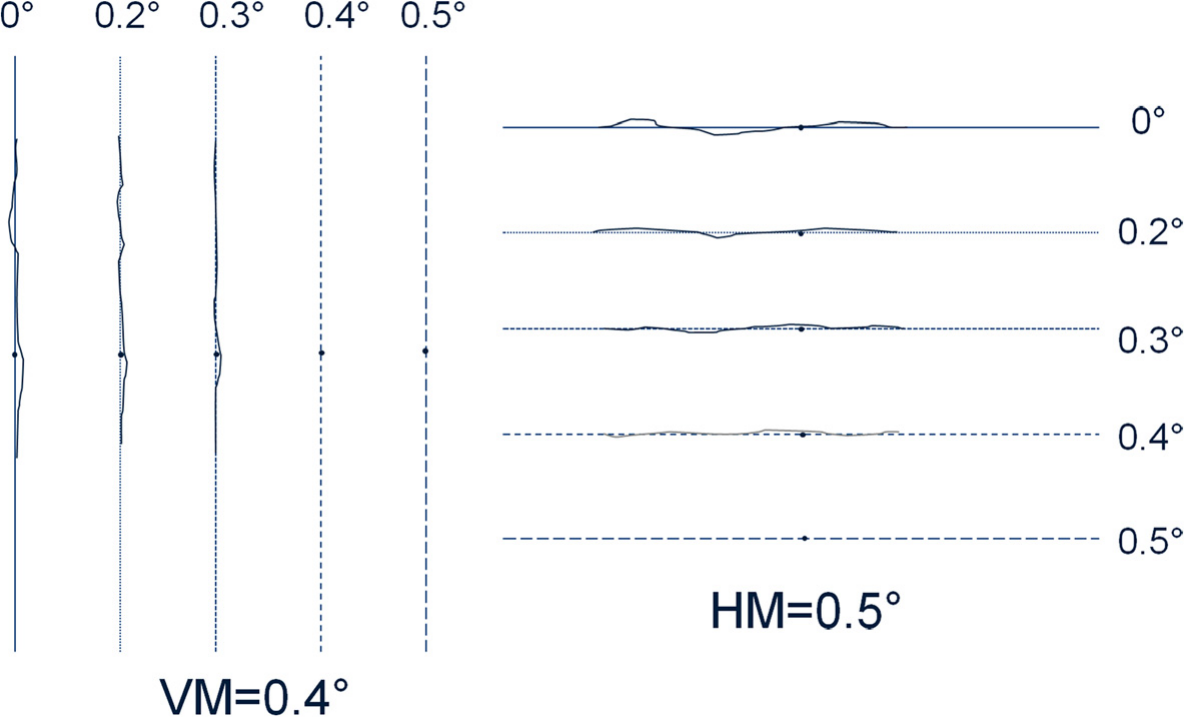
**Example of the result of vertical (VM = 0.4°) (left) and horizontal (HM = 0.5°) (right) M-chart.** Dotted lines are shown to the subject one after another (starting with the solid line - 0°), until the subject recognizes the line as being straight. In this case the vertical solid line was very distorted, following lines with larger dot intervals were recognized as less distorted. Line 0.4° was recognized as straight, thus the vertical metamorphopsia score is 0.4°. After rotating M-charts 90° into the horizontal direction the same procedure was perfomed, until line 0.5° was recognized as straight, thus the horizontal M-chart score is 0.5°.

The examiner presents consecutive dotted lines, starting with a solid line (0°) and the patient has to state whether the presented line is distorted or not. If the patient recognizes the dotted line as being straight, its visual angle is considered to be the metamorphopsia score (Figure 1). M-charts are presented in vertical and horizontal direction (having been rotated by 90°).

As the quantitative parameter of the structural examination the central retinal thickness (CRT) was used. CRT is the distance between the anterior (internal limiting membrane) and posterior (retinal pigment epithelium) highly reflected boundaries of the retina. Additionally, OCT results were assessed qualitatively and classified as serous pigment epithelial detachment, subretinal fluid, cystic retinal oedema or combination of these conditions.

For the present study, one eye of each patient was taken into consideration. The average age of examined patients was 72.0 years (range 50–87 years). The median visual acuity was 0.3 (range 0.1–0.7). All patients underwent intravitreal injection of bevacizumab (1.25 mg) and were examined before and one month after treatment. The injections were performed in a standard manner via pars plana in an operating room [11].

Additionally, 23 age-matched healthy subjects (13 men, 10 women) without retinal pathology were examined as a control group. The median visual acuity was 1.0 (range 0.8–1.0), the average age 68.3 years (range 52–90 years). The average CRT was 265.5 μm (95% CI 256–275 μm).

### Statistical analysis

The Amsler test was considered positive if any blurred lines were reported by the patient. The M-chart result was considered positive if the metamorphopsia score was more than 0° (0.2–2.0°). If it was 0°, it was considered as negative. This assessment was done in regard to vertical and horizontal charts separately and for both methods together – it was considered as positive if one of them was positive, and negative if both were negative. The medians of the vertical and horizontal metamorphopsia score as well as distance visual acuity and CRT before and after bevacizumab injection were compared using the Wilcoxon test. The Spearman’s correlation coefficient was used to assess the relationship between the metamorphopsia, visual acuity and the CRT. Mann–Whitney test was used to assess the relationship between vertical and horizontal M-chart and quantitative results of OCT.

## Results

The rate of the detection of the metamorphopsia (percentage of positive results) of vertical M-chart was 75% before the injection and 56% after the injection, horizontal M-charts - 84% and 56%, both together - 89% and 64%, the sensitivity of Amsler test - 69% and 58% respectively. The vertical metamorphopsia score decreased after the bevacizumab injection in 16 patients (0.2° average); it increased in 7 patients (mean 0.1°); there was no change in 13 patients. The horizontal metamorphopsia score decreased in 22 patients (0.2° average), increased in 3 patients (0.1° average), there was no change in 11 patients.

**Figure 2:**
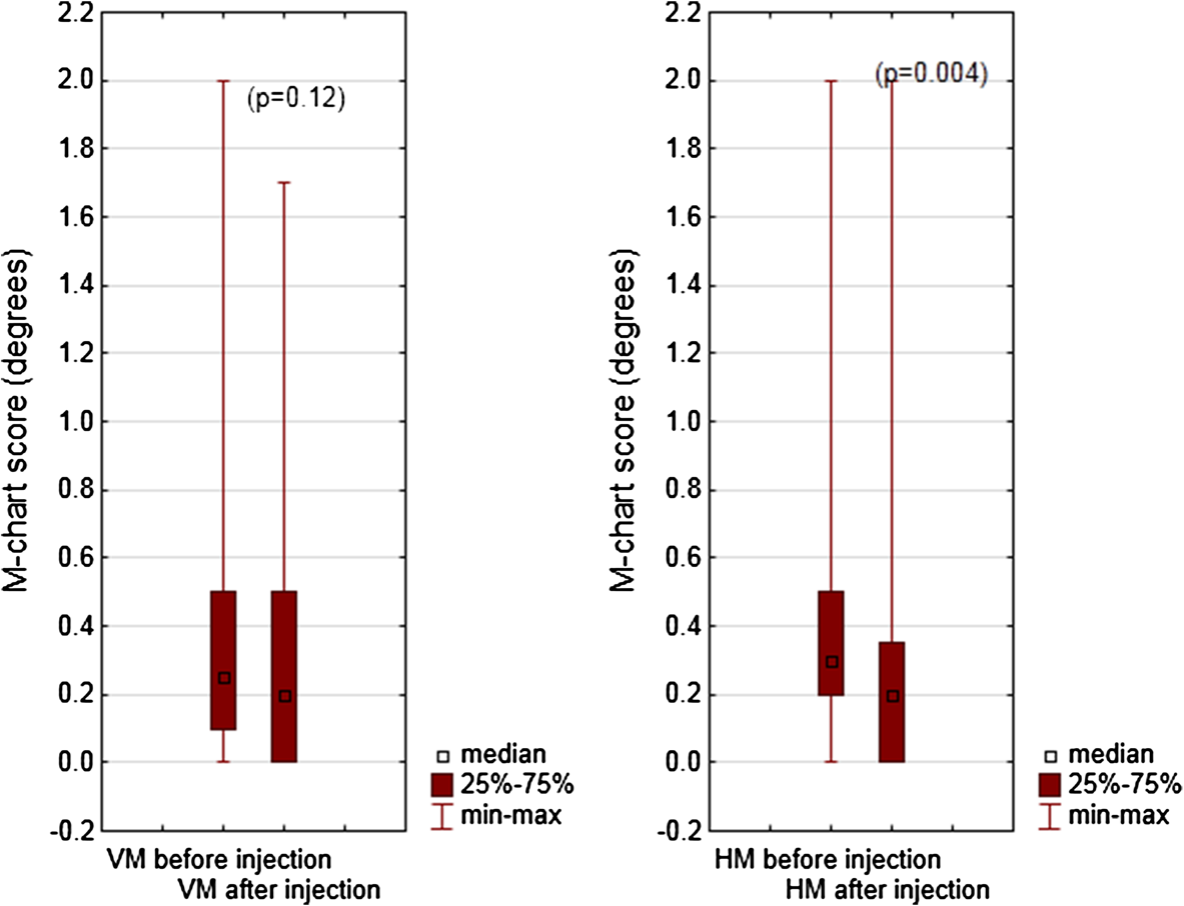
Medians of the vertical - VM (left) and horizontal - HM (right) metamorphopsia score obtained with M-charts before and after the bevacizumab injection.

The median of the vertical metamorphopsia score was 0.25° (range 0–2.0) before the bevacizumab injection and 0.2° (range 0–1.7) after the bevacizumab injection (p = 0.12). The median of the horizontal metamorphopsia score was 0.3° (range 0–2.0) before the bevacizumab injection and 0.2° (range 0–2.0) after the bevacizumab injection (p = 0.004) (Figure 2).

The Amsler grid results improved in 6 patients, deteriorated in 2 patients and remained the same in 28 patients. The visual acuity improved in 17 patients, remained the same in 13 patients and deteriorated in 6 patients. The median distance visual acuity was 0.3 before the bevacizumab injection and 0.35 after the injection (p = 0.008).

The median CRT in OCT was 343.5 μm (95% CI 305.8–381.4 μm) before the injection and 285.4 μm (95% CI 250–320 μm) after the injection (p < 0.01). CRT improved in 33 patients. No relationship was found between the vertical and horizontal metamorphopsia score and the visual acuity (R = −0.2 and R = −0.06, respectively) or with CRT (R = −0.14 and R = −0.001, respectively). The results of OCT were as follows: subretinal fluid and intraretinal cysts in 15 cases, intraretinal cysts only in 13 cases, pigment epithelium detachment in 5 cases, subretinal fluid only in 2 cases and pigment epithelium detachment with subretinal fluid in 1 case. There were no statistically significant differences (p > 0.05) in horizontal and vertical M-charts between above mentioned groups of patients.

All the healthy age-matched subjects revealed no metamorphopsia either in Amsler test or in vertical and horizontal M-charts (median 0°), thus the specificity of both the M-charts and Amsler grid was 100%.

## Discussion

The present study is the first investigation comparing the results of the M-charts and the Amsler grid with OCT results in the assessment of metamorphopsia in patients with wet AMD treated with the intravitreal bevacizumab injections.

It has been demonstrated that the M-charts may be used as a diagnostic tool in the detection of metamorphopsia in AMD patients. The rate of metamorphopsia detection in patients classified to the bevacizumab injection was higher with the M-charts (89%) than with Amsler test (69%). Thus, it seems that the M-charts are superior to the Amsler grid in detecting metamorphopsia in patients with wet AMD. In the current study, it has also been shown that with the M-charts, it is possible to monitor quantitatively the changes in the degree of metamorphopsia over time. The median of the vertical metamorphopsia score decreased from 0.25° to 0.2° after the bevacizumab injection (not significant) and the median of the horizontal metamorphopsia score decreased from 0.3° to 0.2° (significant). The improvement in the meatmorphopsia score after the injection suggests that the M-charts can be used in the assessment of the effectiveness of treatment with intravitreal injections. The M-charts have already been used in the evaluation of the effectiveness of vitrectomy in patients with macular holes [9,12]. Arimura and colleagues have shown that after vitrectomy visual acuity improved in 14 of 22 patients and metamorphopsia scores improved in 19 of 22 patients [9].

The best current practice involves using the Amsler charts for detecting and monitoring metamorphopsia in AMD. However, it has already been reported that the Amsler test has poor sensitivity [12]. The sensitivity of the Amsler chart in AMD varies between 9% in early stages to 34% in choroidal neovascularisation [13]. In a study comparing the Amsler grid with microperimetry, the sensitivity of the Amsler chart was only 56%; in patients with small scotomas, the false negative rate was found to be 77% [14]. There are many possible explanations for this high false-negative rate. Firstly, the filling-in phenomenon across pathological scotomas [14]; secondly, the use of a preferred retinal locus away from the scotoma boundary [15]; moreover, probably, the averaging of crowded stimuli [16]. Moreover, the results of the Amsler charts are only descriptive; they are neither precise nor reproducible. Thus the Amsler chart alone does not seem to be sufficient for the metamorphopsia detection and for monitoring the changes over time.

The M-charts provide more information than the Amsler chart in regard to the horizontal and vertical lines. It has been found that the decrease of metamorphopsia was significant for horizontal lines only. The difference between horizontal and vertical metamorphopsia has been already observed by Amsler [17]. Matsumoto found [7] that metamorphopsia is more severe in horizontal than in vertical lines in patients with epiretinal membranes. He proposed that the abovementioned fact may be explained by the differences of human visual sensitivities between horizontal and vertical visual fields [18].

Up to now, some modifications have been made to the traditional Amsler chart, such as threshold Amsler testing with cross-polarizing filters reducing the luminance of the grid [19]. It has been shown that it increased the detection rate in patients with diabetic retinopathy [20]. An alternative test used as a replacement for the Amsler grid is the Macular Computerized Psychophysical Test introduced in 2003 [13]. In this test, a virtual line composed of dots (white dots on a black background, maximal contrast on the computer screen) is flashed across different macular loci to a perifoveal radius of 7 degrees. The patient is asked to report any blurring or gaps within the line. The commercial form of this method is known as the Preferential Hyperacuity Perimetry (PHP). In this test, a single straight dotted line with a few dots out of alignment is flashed across different macular loci over a macular field of 14° × 14°. The patient uses a stylus to touch the screen where he had experienced a distortion in the line. Any distortion perceived by the patient is automatically recorded and analyzed, and a macular map showing the area of distortion and the intensity of metamorphopsia is displayed. The authors reported the superiority of this test over the Amsler grid in patients with AMD [21]. PHP has been also used in the assessment of metamorphopsia in AMD patients treated with ranibizumab [22]. The improvement of metamorphopsia score was observed after 6 months after the injection. In a study comparing PHP with the M-charts published by Arimura [23], the M-charts showed better sensitivities than PHP in both ERM (89% vs. 42%) and AMD (74% vs. 68%). However, PHP is complicated and rather expensive, thus it is not widely used in clinical practice.

In order to be deemed trustworthy, a diagnostic method should have an acceptable level not only of sensitivity, but also of specificity. In the current study, no healthy person has seen metamorphopsia either with the M-charts or the Amsler test. Thus, the specificity of both methods was 100% and there were no false-positive results. However, in a study performed by Loewenstein, the Amsler chart has been shown to indicate the presence of scotomas in about 2% of control subjects without any scotoma [13]. In a study comparing Amsler grid with PHP, there was a better specificity of PHP - it was 100% vs. 71% with the Amsler grid in healthy controls [23].

Interestingly, no significant relationship was found between the degree of metamorphopsia and the visual acuity in our group of patients with AMD. The same finding was described by Matsumoto [6] in a group of patients with metamorphopsia due to epiretinal membrane. Thus, metamorphopsia seems to be independent of the visual acuity, being another clinical aspect of the visual function. Metamorphopsia is not a simple symptom and it consists of several kinds of components. In particular, there are several kinds of frequency components of distortion in metamorphopsia. Moreover, in the present study, no relationship was found between the degree of metamorphopsia and CRT as well as morphological changes in OCT. A longer follow-up is needed to determine if M-charts can be used to identify the cases with complete resolution of metamorphopsia, the cases with persistent metamorphopsia and the cases with recurrence of metamorphopsia associated with CNV recurrence.

## Conclusions

The study concludes that the M-charts are a simple, fast, easy and cheap method for the quantification of metamorphopsia that can be used even at home for self-assessment. The present research has shown that the M-charts are superior to the traditional Amsler charts, as they detect metamorphopsia even if the Amsler chart is normal. The M-charts provide additional information in regard to the visual function independent from visual acuity and OCT results and can be used in the quantitative assessment of the progression of metamorphopsia in patients suffering from wet AMD treated with bevacizumab injections.

## Competing interests

The authors declare that they have no competing interests.

## Authors’ contributions

KN-design of the study, analysis and interpretation of the data, writing the manuscript, AO-acquisition of the data, performing examinations, AB-statistical analysis, AG-critical analysis of the results, revision of the manuscript; PK and RR-acquisition and analysis of the data and revision of the manuscript for important intellectual content, general supervision. KK, AJ and RM were involved in drafting the manuscript and revising it critically for important intellectual content. All authors read and approved the final manuscript.

## Pre-publication history

The pre-publication history for this paper can be accessed here:

http://www.biomedcentral.com/1471-2415/13/13/prepub
